# Monitoring Carbon in Electron and Ion Beam Deposition within FIB-SEM

**DOI:** 10.3390/ma14113034

**Published:** 2021-06-02

**Authors:** Nicholas T. H. Farr, Gareth M. Hughes, Cornelia Rodenburg

**Affiliations:** 1 Department of Materials Science and Engineering, Sir Robert Hadfield Building, University of Sheffield, Mappin Street, Sheffield S1 3JD, UK; c.rodenburg@sheffield.ac.uk; 2 Department of Materials, University of Oxford, Parks Road, Oxford OX1 3PH, UK; gareth.hughes@materials.ox.ac.uk

**Keywords:** carbon contamination, carbon surface analysis, characterisation, focused ion beam microscopy, secondary electron emission, secondary electron hyperspectral imaging, secondary electron spectroscopy

## Abstract

It is well known that carbon present in scanning electron microscopes (SEM), Focused ion beam (FIB) systems and FIB-SEMs, causes imaging artefacts and influences the quality of TEM lamellae or structures fabricated in FIB-SEMs. The severity of such effects depends not only on the quantity of carbon present but also on its bonding state. Despite this, the presence of carbon and its bonding state is not regularly monitored in FIB-SEMs. Here we demonstrated that Secondary Electron Hyperspectral Imaging (SEHI) can be implemented in different FIB-SEMs (ThermoFisher Helios G4-CXe PFIB and Helios Nanolab G3 UC) and used to observe carbon built up/removal and bonding changes resulting from electron/ion beam exposure. As well as the ability to monitor, this study also showed the capability of Plasma FIB Xe exposure to remove carbon contamination from the surface of a Ti6246 alloy without the requirement of chemical surface treatments.

## 1. Introduction

Scanning electron microscopes (SEM) have established themselves as indispensable tools within laboratories across the world and have supported diverse research projects undertaken within many scientific disciples since their development in the 1950s. During this time many innovative new developments, both in operation and construction, have ensured that the SEM is still an essential technique in an increasingly broad range of scientific applications [1]. The availability of enhanced beam control, emission detectors, and improved sample preparation options together with the ability to integrate the data output of the SEM with external processing and analysis facilities provides confidence that the SEM will maintain its established position well into the future [2,3].

The absence of ultra-high vacuum sample chambers in many SEMs and Focused Ion Beam (FIB)-SEMs leaves them exposed to potential contamination from hydrocarbon molecules. Previous research has shown that low material purity in FIB nano fabricated structures results from the incomplete dissociation of gas precursor molecules or volatile residual species present in vacuum chambers, leading to high residual percentages of deposited carbon [4]. It is well understood that hydrocarbon molecules can readily adsorb onto the surface of many target sample types where subsequent exposure to the primary beam results in their decomposition into amorphous carbon, accumulating deposits that reach several nanometres in thickness. This can lead to incorrect reporting of critical dimensions [5], masking doping contrast [6], and increased thickness of otherwise electron transparent areas in Transmission electron microscopy (TEM) specimens [7] or contamination of atom probe tips [8]. Carbon contamination built up during SEM energy-dispersive X-ray spectroscopy (EDX) can be problematic if the effect is not considered in the design of localised corrosion experiments and coatings research and may lead to erroneous conclusions; for example, in the inspection of aluminum alloy 2024-T3 (AA2024-T3) which is widely used in the aircraft industry [9]. Therefore, many SEMs and FIB-SEMs are equipped with plasma cleaners to minimise carbon deposits. To evaluate the effectiveness and necessary plasma cleaning times, contamination monitoring is required. Monitoring of residual gas analysis was carried out in this context [10]. While this delivers information about the nature of contamination sources, it does not provide information about the localized contamination built up during imaging or fabrication. Local build up and reduction of up to 1000 nm thick contamination windows was monitored using nanoflight^®^ SEM movies [11]. However, nanoflight requires extensive hardware as it relies on a multi-detector system which is still ‘a project under construction’ [12].

In some circumstances, an intentional electron beam induced carbon deposition (EBID) from the residual vacuum is exploited to protect surface features prior to ion beam exposure and increase the success rate of TEM lamellas prepared by FIB milling in semiconductor related failure analysis [13]. This technique is also used to protect areas of freshly ion beam thinned TEM lamella from corrosion [14]. In all cases, apart from the deposition parameters (e.g., beam current, electron beam energy, etc.) a detailed analysis of the achieved EBID is not undertaken. Therefore, it is not clear if and how the EBID parameters recommended for one specific FIB-SEM instrument could be translated and utilised in different FIB-SEM instruments. FIB-SEMs are not only widely used for TEM lamella preparation, but also for creating Microelectromechanical systems and photonic meta materials through ion beam deposition. In these applications the carbon is always present in different forms of bonding. Ion beam induced deposition (IBID) can prepare delicate and high aspect ratio three-dimensional nanostructures with excellent mechanical strengths [15]. Advances in FIB deposition has allowed the production of nanostructures and devices with a broad range of applications including micro/nano electromechanical systems [16]. However, the purity of the deposited material is not absolute as carbons species are always retained in the deposited film. For FIB structures, such as nanoconductors, there is a requirement to monitor and remove contamination, improving the conductivity of the deposited metal [16]. Similarly, organic contaminants deposited during FIB fabrication have also been shown to lower the Young’s modulus of three-dimensional microstructures [17]. The impact carbon contamination has on FIB structures is highly dependent on both the material and conditions of carbon contamination.

Varying IBID deposition conditions can form various forms of carbon species (amorphous carbon, graphite, diamond, and diamond like carbon (DLC)) [18]. Such carbon deposited in IBID can strongly affect the mechanical [19] and photonic [4] properties of FIB fabricated structures [20], but differences in carbon species are not routinely determined during the IBID deposition process. The ability to monitor different forms of carbon contamination during the deposition process is still yet to be established. The most obvious reason for this is a lack of suitable characterisation tools is the combination of multiple requirements: high surface sensitivity (akin to X-ray photoelectron spectroscopy (XPS), high spatial resolution (akin to Auger electron spectroscopy) [21], and the ability to identify the carbon bonding present using low beam energies (reducing sample modification) without the need for an Ultra High Vacuum (UHV) [22,23]. All of these requirements may be fulfilled by Secondary Electron (SE) spectroscopy. SE spectroscopy is not a new concept and has a research history as long as the SEM [24]. However, it is only relatively recently through technology developments in detection instrumentation, signal processing, and imaging technologies that it has become the focus of new SEM capabilities [22,25,26]. Through-the-Lens Detectors (TLDs) are installed on many available SEMs and FIB-SEMS, providing a low pass SE collection facility at low primary electron beam energies and currents [27,28]. Some TLDs, certainly the ELSTAR Column (FEI, Thermo Fisher), enable the compilation of stacks comprising of SE images taken from the same region of interest with each image formed by SES of different energy ranges, enabling secondary electron hyperspectral imaging (SEHI). From the SEHI stack, SE spectra (SES) can be derived or specific energy ranges utilised to compile surface chemistry maps down to the nanoscale. Diverse applications including chemically mapping semi-crystalline polymers, identifying nanostructure variations within natural materials, and molecular orientation analysis of organic electronic devices, have all demonstrated the benefits of the SEHI chemical mapping [27,29,30,31]. Carbon EBID has previously been investigated using SEHI [30], but IBID has not had the same level of analysis applied. In this study, we consider SES/SEHI applications for both IBID and EBID.

## 2. Materials and Methods

Sample preparation:

Highly Oriented Pyrolytic Graphite (HOPG) samples (Agar Scientific Mosaic) were prepared as either fresh or aged. Fresh surfaces are prepared by revealing a surface layer by the application of mechanical exfoliation and are required to be loaded into the instruments sample chamber within a 1 min time period. In contrast, aged surfaces have no special surface preparation and are exposed to atmospheric conditions for substantial periods prior to observation.

Plasma FIB exposure:

A surface of Ti6246 alloy (Al 6%, Sn 2%, Zr 4%, Mo 6%, Ti bal.) was finished to 1200 grit and mounted for FIB SEM analyses. An area of 10 × 10 µm was chosen for exposure with the Xe^+^ focused ion beam on the Helios G4-PFIB system. An initial SEHI data set was acquired on the virgin (unexposed) surface. The surface was then exposed to a 10 µm × 10 µm box pattern using the standard Si application file (ThermoFisher Scientific/FEI) at an accelerating voltage of 30 kV and an ion beam current of 1 nA. Each exposure was set to 20 s with a dose calculation of 0.2 nC/µm^2^. After each exposure, a SEHI data set was acquired over the central (10 × 10) µm region and then an expanded (20 × 20) µm field of view, to compare the exposed region with unexposed exterior.

Conventional low KV Imaging:

FEI Helios Nanolab G3 UC (FEI Company, Hillsboro, OR, USA) and Thermo Fisher Scientific Helios G4 CXe PFIB DualBeam (Thermo Fisher Scientific, Eindhoven, The Netherlands) microscopes were employed for surface morphology observations of HOPG and Ti6246. In contrast to established SEM analysis practice, neither the HOPG nor the Ti6246 samples were treated with a conductive coating through deposition. A low (1 kV) accelerating voltage to gether with typical chamber vacuum pressures in the range of 10^−6^ mbar using a working distance of 4 mm were chosen to avoid sample damage through surface charging. For low magnification SE images, an Everhart-Thornley Detector (ETD) was selected and for high magnification SE images a TLD was selected.

SEHI data collection and processing:

SES generation was performed on both HOPG and Ti6246 using the Helios Nanolab G3 UC microscope and Helios G4 CXe PFIB by applying consistent operating conditions of 1 kV (monochromated) and 50 pA immersion mode (mode II/UHR). These microscopes are capable of providing ultrahigh resolution images at voltages <1 kV. To ensure that images were taken of the actual material surface, no conductive coating deposition was applied to the samples in contrast to typical SEM analysis practice. A typical vacuum pressure of ~10^−6^ mbar, working distance of 4.0 mm, and an accelerating voltage of 1 kV were applied in immersion mode. The collection of SES of different energy ranges was enabled through the adjustment of the mirror electrode voltage (MV) together with a tube bias setting of 150 V. Stepping the MV in a range of −15 V and 15 V (energy range of −0.7 to 12.7 eV) was achieved through the use of an automatic iFast collection recipe [32]. Every image was captured at a frame interval of 0.5 s and an MV step size of 0.5 V which corresponds to ~0.2 eV electron energy step size. Image processing was undertaken using Fiji Image J software (Image J2, open-source). The SES were obtained by differentiating the captured S curves. Isolating components of interest were achieved by performing a 6 component analysis of the image stacks through non-negative matrix factorisation (NNMF) [33,34].

## 3. Results and Discussion

### 3.1. Understanding Electron Beam Deposition by Analysing Spatial Variations

**Figure 1:**
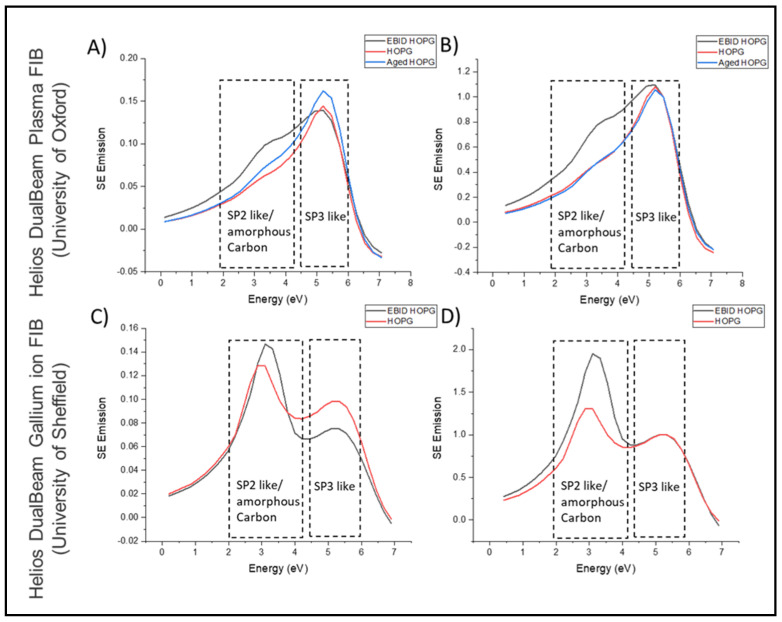
(**A**) SES for EBID HOPG, HOPG, and Aged HOPG collected in a Helios DualBeam Plasma FIB. (**B**) SES normalised to 5.2 eV-sp^3^ bonding peak for EBID HOPG, HOPG, and Aged HOPG collected in a Helios DualBeam Plasma FIB. (**C**) SES for EBID HOPG, HOPG, and Aged HOPG collected in a Helios DualBeam Gallium FIB. (**D**) SES normalised to 5.2 Ev-sp^3^ bonding peak for EBID HOPG, HOPG, and Aged HOPG collected in a Helios DualBeam Gallium FIB.

Figure 1 presents the comparison of HOPG SES collected in two separate Helios instruments. Here, the collection of SES from HOPG surfaces verifies SE peak positions, and acts as an initial calibration. From the collected SES, it is observed that both instruments expressed SE peak emissions at the same energy values. Two clear peaks were displayed by both instruments in the energy regions of 2–4.2 eV and 4.6–6 eV. Previous studies, which have generated SES of HOPG, confirm these findings and have shown 2–4.2 eV peaks are formed as a result of sp^2^ and amorphous carbon contamination and that the 4.6–6 eV peaks are related to sp^3^ bonding [30].

Expected SE emission differences between the two SES plots appear in the peak intensities previously identified as sp^2^ and sp^3^ carbon bonding [27,30,35]. This initial baseline SES collected is useful not only to monitor carbon, but also to understand the cleanliness of an SEM chamber and what forms of contaminant are present. As previous studies have shown, emission in the sp^2^ energy range, an amorphous carbon contamination (ACC) region in HOPG, is an indication of EBID related contamination found within the SEM chamber [30,36]. Surface contamination forms will be influenced and characterised by the different chamber environments that occur in the two microscopes. This form of contamination (ACC) is highly dependent on the samples analysed and any FIB applications that have been performed in the chambers. Specifically, two forms of contamination are expected: ambient air contamination of hydrocarbons (sp^2^) [37] and EBID contamination of carbonaceous species (sp^2^/sp^3^) during SEM analysis operation. Both these forms of contamination have the potential to deposit on the native surface of a sample thereby reducing image resolution and compromising the effectiveness of sample analysis [38]. To reduce the potential of an air contaminated HOPG sample surface affecting the resulting SES, the HOPG was subjected to a process of exfoliation which reveals “fresh” layers on the top surface. This process ensures that the SES spectra collected from samples within different SEM chambers with dissimilar environments can differentiate the emission stemming from amorphous carbon formation, observed in the contamination spectra compared to that of the initial fresh HOPG SES spectra. 

**Figure 2:**
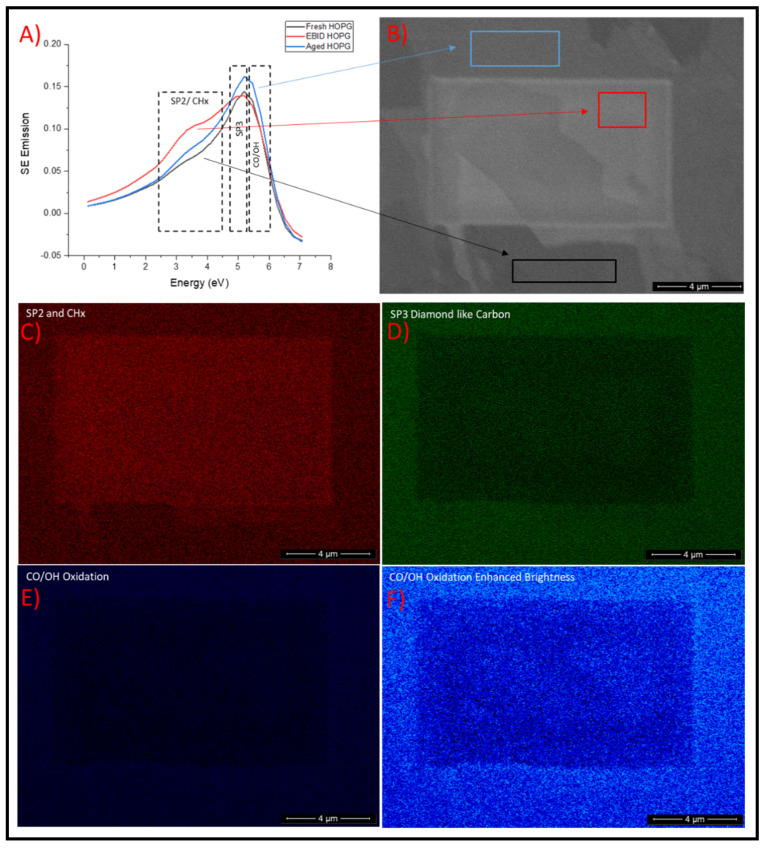
(**A**) Secondary electron spectra for EBID HOPG, HOPG and Aged HOPG collected in a Helios DualBeam Plasma FIB. (**B**) Presents an SE image of the region used for SES collection. (**C**) Presents SE chemical mapping of sp^2^/CHx. (**D**) Presents SE chemical mapping of sp^3^ bonding. (**E**) Present SE chemical mapping of CO/OH bonding. (**F**) Presents contrast enhanced SE chemical mapping of CO/OH bonding.

To better understand these forms of carbon contamination, and the effect of sustained EBID, SES was collected from various areas of interest on an HOPG surface within the Helios DualBeam Plasma FIB. Figure 2A shows the resulting SES spectra from the various regions identified within Figure 2B. The three regions are termed EBID HOPG, HOPG and Aged HOPG. As the Figure 2B shows, the EBID HOPG spectrum was taken from within a typical EBID window formed on a freshly exfoliated HOPG’s surface by scanning the area with the electron beam for 60 s. The HOPG spectrum was collected in a region outside this scan window. The Aged HOPG spectrum stems from a grain that appears much brighter than most of the freshly exfoliated HOPG. Therefore, it is assumed that this is a grain of HOPG which had not been cleaved away completely during the exfoliation procedure. All three regions showed peak emissions in the two ranges highlighted above in Figure 1, which is consistent with previous studies. EBID HOPG exhibited a larger emission for ACC build up than that of Aged HOPG and the exfoliated HOPG. Aged HOPG displayed greater ACC than that of exfoliated HOPG, and also a greater sp^3^ peak than that of both the other regions.

It was previously shown that NNMF component analysis of SEHI image stacks can be conducted to provide chemical maps. Here SEHI stacks captured from the HOPG surfaces underwent NNMF component analysis which identified various spectral components (Supplementary Materials Figure S1) with peak positions and respective assignments to functional groups based on previous work [27,30,35]. Segmentation based on these components then formed the basis for the chemically resolved SEHI stacks displayed. This process has previously been applied to other organic materials; however, this is the first time it has been shown to map carbon bonding within HOPG. From the SEHI stacks produced (Figure 2C) it is clear that a strong emission within the EBID window is present for sp^2^ and ACC. This result further indicates that EBID can contribute to ACC deposition. Comparing Figure 2C SEHI stack to Figure 2D suggests that ACC has the ability to prevent the emission of sp^3^ surface aged contamination by replacing it with ACC. 

Lastly from SEHI chemical mapping Figure 2E was produced via the uncovering of a component from NNMF in a range previous considered to be emissions resulting from the inclusion of oxygen containing functionalities [27]. The primary factor responsible for carbon surface evolution is the adsorption of water which significantly affects the properties of the surface. The emission signal displayed in the original CO/OH SEHI map (Figure 2E) was initially difficult to clearly visualise therefore an enhanced brightness map was produced and is given in Figure 2F. Here it is noticeable that oxidation of HOPG appears to be concentrated in regions of Aged HOPG and is covered in part by EBID contamination. Greater emission is visible in the top right hand corner of the image, which in the original SE images provided in Figure 2B shows as older grains of HOPG which have not been fully exfoliated. This in plausible as slight oxidation or moisture build up would be expected to occur on an older HOPG grain which has been exposed to ambient conditions during long term sample storage.

### 3.2. Understanding Electron Beam Deposition by Analysing Spatio-Temporal Variations

**Figure 3:**
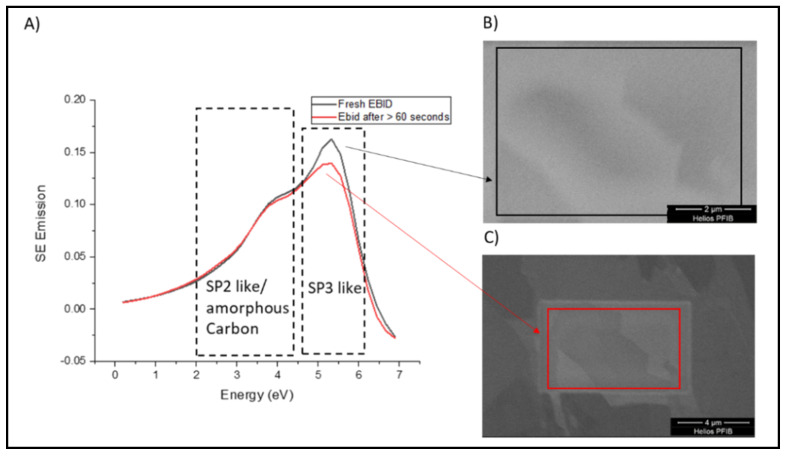
(**A**) Secondary electron spectra for Fresh EBID and EBID after >60 s collected in a Helios DualBeam Plasma FIB. (**B**) Presents an SE image (10 µm HFW) of the Fresh EBID region used for SES collection. (**C**) Presents an SE image (20 µm HFW) of the same region given in B however after >60 s which was then used for SES collection.

From the maps in Figure 2 is clear that there is not a single form of contamination but EBID starts a process of higher ACC deposition. To observe this effect further SEHI stacks were collected and SES (Figure 3A) extracted as follows: first stacks were collected from a 10 µm wide field of view (HFW) (Figure 3B); then this was increased to a 20 µm wide HFW (Figure 3C). This approach was chosen to allow for SES to be collected from different regions including at the 10 µm HFW with EBID contamination for a specified electron beam exposure time. This enabled the extraction of SES spectra of fresh EBID at 10 µm HFW followed by SES spectra of EBID after 60 s during the collection of the 20 µm HFW stack. The SES Spectra comparison of these two time points is presented in Figure 3A. 

The most notable difference is seen with the increase in sp^3^ carbon emission of fresh EBID compared to EBID after 60 s. The ability of EBID to create sp^3^ emission has previously been highlighted in a range of analytical techniques including SES [30]. Chemisorption of hydrogen is put forward as the principal mechanism of contamination which is anticipated to initiate at irregularities on the graphite surfaces [39]. The chemisorption of hydrogen increases surface electron emission through transforming the work function of the HOPG surface leading to deformation and the conversion to sp^3^- like distorted bonds of sp^2^ bonds [40]. Of particular interest is that this emission range is not only reduced after the 60 s aging (See Figure 3) but it also reveals that the sp^2^ amorphous carbon build up appears to slightly increase which perhaps indicates a mechanism by which sp^3^ carbon emission is the first to form on the surface of HOPG in response to EBID but then as a consequence creates changes in the surface energy which then promotes the rise of amorphous carbon attachment.

### 3.3. Understanding Carbon Modification by Xe-Ion Beam Exposure

**Figure 4:**
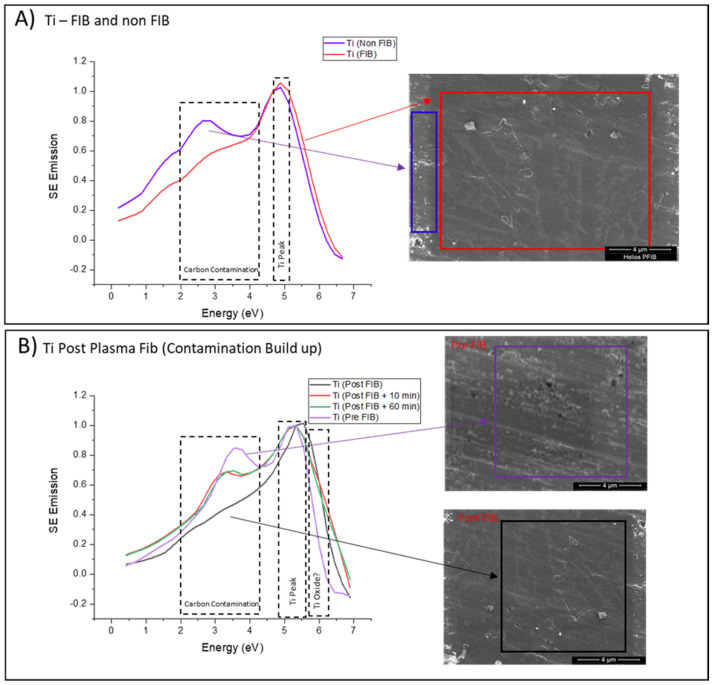
(**A**) SES and accompanied SE images of the SES collection regions for Ti and Ti after FIB collected in a Helios DualBeam Plasma FIB. (**B**) SES and accompanied SE images of the SES collection regions for Ti and Ti after FIB, at various time points, collected in a Helios DualBeam Plasma FIB.

Figure 4A shows that SES spectra of Ti6246 alloy (Ti) pre and post Plasma FIB Xe ion exposure. Plasma FIB was used in this instance to create a well-defined clean area within which surface contamination is removed via surface sputtering. This “cleaned” area was then used to evaluate the buildup of ACC over two time points: 30 and 60 min after cleaning. In order to observe changes within ACC, subsequent SES spectra was scaled to that of the Ti alloy peak (5.2 eV). It is noted that for Auger spectra, previous studies have isolated peak ranges of 4.9–5.3 eV for Ti (0001) with oxidation creating peaks around 5.5–6.2 eV, these are comparable to oxygen containing functionality peaks observed in SES [41,42].

Two clear differences arise within the SES obtained pre and post FIB (Figure 4A,B). The first being the reduction of the peak situated between 2–4.3 eV, previously associated with primary surface ACC, post FIB exposure. The signal signature for this form of contamination is greatly reduced as the FIB window is created, and as post FIB SES indicate at the time points, it is observed that this contamination does start to return to the surface of the materials as the sample undergoes conventional SEM imaging. This finding revealed that as well as the ability of SES to monitor ACC, Plasma FIB Xe exposure has the capability to remove carbon contamination from the surface of Ti without the requirement for chemical surface treatments [23]. The removal of carbon contamination is seen as an advantage for IBID fabrication, notably for nanoconductors to improve the conductivity of the deposited material and to increase the tensile elasticity of FIB fabrication microstructures [17]. 

The second SE spectral difference is displayed between 5.4 and 6.1 eV. SE spectra collected previously have shown the ability to isolate oxygen functionalities within organic materials between the SE emission range of 4.1–5.5 eV. Therefore, this indicates the region of this emission could be in response to Ti oxides. Such findings of surface oxidation (and the presence of nitrogen) as a result of plasma-FIB exposure have recently been identified on a TiAl alloy [43]. Procedures to reduce carbon contamination (such as post-deposition irradiation) have previously used oxygen to form volatile species (CO and CO_2_) to reduce carbon content. Despite the effective carbon removal, post-deposition treatments increase oxygen traces which have been detected in the resulting nanostructure composition. SES shows to have the ability to monitor oxygen species and could be a useful tool to further evaluate post deposition carbon removal treatments as well as monitor oxidation [4]. However, future work is required to better understand SE peak emission of inorganic oxides. As this form of Ti alloy is expected to contain some surface oxidants, the explanation for the existence of this peak emission could either be the result of FIB removing contamination after aged surface oxidation or it is considered that post FIB the Ti surface energy is changed which results in a fresh reactive surface which not only attracts the reformation of surface contamination, but also slightly increases surface oxidation. 

## 4. Conclusions

This study highlights SES and SE chemical mapping abilities to monitor and evaluate various forms of sample contamination within an SEM chamber from evidence taken from two different DualBeam SEM instruments. Results from the study also confirmed that SE chemical mapping has the capacity to chemically map surface contamination in both organic and non-organic material systems. As a consequence of being able to monitor localised carbon contamination, it was shown that Plasma FIB Xe exposure has the capability to remove carbon contamination from the surface of Ti6246 alloy without the use of chemical surface treatments. The importance of understanding the surface structure and chemical mapping of materials prior to FIB building or SEM imaging is a well-known requirement that has been substantiated by the study’s discussions based on captured SES. SES is available for FIB-SEMs fitted with a TLD detector, which makes the opportunity for users to adopt this methodology easily accessible without the requirement for any additional instrumental extensions.

## Data Availability

Secondary Electron Spectral Acquisition iFAST Script (Automatic) available—https://doi.org/10.15131/shef.data.14535993.v1 (accessed on 4 May 2021).

## Supplementary Materials

The following are available online at https://www.mdpi.com/article/10.3390/ma14113034/s1.
