# Supplementary material for: Monitoring Carbon in Electron and Ion Beam Deposition within FIB-SEM
Source: Materials (Basel). 2021 Jun 2;14(11):3034. doi: 10.3390/ma14113034 (PMC8199708; doi:10.3390/ma14113034)
Supplement: Supplementary file 1 [file materials-14-03034-s001.zip › materials-1230000-supplementary.pdf]

materials

MDPI

Article

Monitoring Carbon in Electron and Ion Beam Deposition within FIB-SEM

Nicholas T.H. Farr [1,*], Gareth M. Hughes [2] and Cornelia Rodenburg [1]

1 Department of Materials Science and Engineering, Sir Robert Hadfield Building, Mappin Street, University of Sheffield, Sheffield S1 3JD, UK; c.rodenburg@sheffield.ac.uk
2 Department of Materials, University of Oxford, Parks Road, Oxford OX1 3PH, UK; gareth.hughes@materials.ox.ac.uk
* Correspondence: n.t.farr@sheffield.ac.uk

Abstract: It is well known that carbon present in scanning electron microscopes (SEM), Focused ion beam (FIB) systems and FIB-SEMs, causes imaging artefacts and influences the quality of TEM lamellae or structures fabricated in FIB-SEMs. The severity of such effects depends not only on the quantity of carbon present but also on its bonding state. Despite this, the presence of carbon and its bonding state is not regularly monitored in FIB-SEMs. Here we demonstrated that Secondary Electron Hyperspectral Imaging (SEHI) can be implemented in different FIB-SEMs (ThermoFisher Helios G4-CXe PFIB and Helios Nanolab G3 UC) and used to observe carbon built up/removal and bonding changes resulting from electron/ion beam exposure. As well as the ability to monitor, this study also showed the capability of Plasma FIB Xe exposure to remove carbon contamination from the surface of a Ti6246 alloy without the requirement of chemical surface treatments.

Keywords: carbon contamination; carbon surface analysis; characterisation; focused ion beam microscopy; secondary electron emission; secondary electron hyperspectral imaging; secondary electron spectroscopy

Citation: Farr, N.T.H.; Hughes, G.M.; Rodenburg, C. Monitoring Carbon in Electron and Ion Beam Deposition within FIB-SEM. *Materials* **2021**, *14*, 3034. https://doi.org/10.3390/ma14113034

Academic Editor: Julio Ramírez-Castellanos

Received: 5 May 2021
Accepted: 28 May 2021
Published: 2 June 2021

Publisher's Note: MDPI stays neutral with regard to jurisdictional claims in published maps and institutional affiliations.

Copyright: © 2021 by the authors. Licensee MDPI, Basel, Switzerland. This article is an open access article distributed under the terms and conditions of the Creative Commons Attribution (CC BY) license (http://creativecommons.org/licenses/by/4.0/).

Supporting Information

In order to obtain images from the corresponding spectral components a non-negative matrix factorisation (NNMF) was used to isolate various components between 0–7 eV. Figure 2C–F in the main manuscript displays SEHI stacks of three components from HOPG surfaces that can be matched to the peak allocation shown in Figure S1 compared to that of the differentiated SE spectra`s collected. Of specific interest, one component consisted of a peak 3.6 eV highlighted that was previously identified as SP2 and CH vibrations. Additionally, a component at 5 eV associated with SP3 bonding is observable, together with a peak evident around 5.5 eV related OH bonding. Here SEHI demonstrates that it is possible to map carbon bonding on the surface of HOPG using the components generated from NNMF.

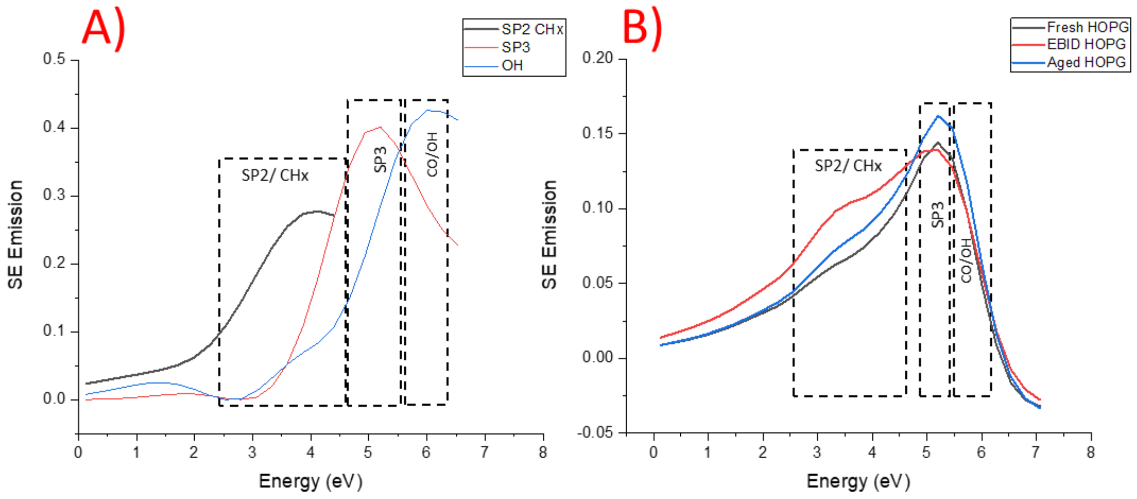

Figure S1. (**A**) Non negative matrix factorisation (nnmf) multivariate analysis of HOPG. Isolating three components (SP2, SP3 and OH bonding). (**B**) Secondary electron spectra for EBID HOPG, HOPG and Aged HOPG collected in a Helios DualBeam Plasma FIB.
